# Retraction Statement: Comparative molecular docking and molecular‐dynamic simulation of wild‐type‐ and mutant carboxylesterase with BTA‐hydrolase for enhanced binding to plastic

**DOI:** 10.1002/elsc.202270113

**Published:** 2022-11-23

## Abstract

**Retraction: ‘Comparative molecular docking and molecular‐dynamic simulation of wild‐type‐ and mutant carboxylesterase with BTA‐hydrolase for enhanced binding to plastic**’, by Fatana Lameh, Abdul Qadeer Baseer, and Abubakar Garba Ashiru, Eng Life Sci. 2021; 13‐29: The above article, published online on 15 November 2021 in Wiley Online Library (https://doi.org/10.1002/elsc.202100083), has been retracted by agreement between the authors, the journal's Editors in Chief, Prof. Dr. Ralf Takors and Prof. Dr. An‐Ping Zeng, and Wiley‐VCH GmbH.

The retraction has been agreed because the copyright owner, Universiti Teknologi Malaysia, does not consent to publication of the research.

**Retraction: ‘Comparative molecular docking and molecular‐dynamic simulation of wild‐type‐ and mutant carboxylesterase with BTA‐hydrolase for enhanced binding to plastic**’, by Fatana Lameh, Abdul Qadeer Baseer, and Abubakar Garba Ashiru, Eng Life Sci. 2021; 13‐29: The above article, published online on 15 November 2021 in Wiley Online Library (https://doi.org/10.1002/elsc.202100083), has been retracted by agreement between the authors, the journal's Editors in Chief, Prof. Dr. Ralf Takors and Prof. Dr. An‐Ping Zeng, and Wiley‐VCH GmbH.

The retraction has been agreed because the copyright owner, Universiti Teknologi Malaysia, does not consent to publication of the research.

